# Exploring lipin1 as a promising therapeutic target for the treatment of Duchenne muscular dystrophy

**DOI:** 10.1186/s12967-024-05494-z

**Published:** 2024-07-16

**Authors:** Abdulrahman Jama, Abdullah A. Alshudukhi, Steve Burke, Lixin Dong, John Karanja Kamau, Brooklyn Morris, Ibrahim A. Alkhomsi, Brian N. Finck, Andrew Alvin Voss, Hongmei Ren

**Affiliations:** 1 Department of Biochemistry and Molecular Biology, Wright State University, 3640 Colonel Glenn Hwy., Dayton, OH 45435-0001 USA; 2 Department of Biological Sciences, Wright State University, Dayton, OH USA; 3 Mumetel LLC, University Technology Park at IIT, Chicago, IL USA; 4 Department of Medical Laboratories, College of Applied Medical Sciences, Qassim University, Buraydah, Saudi Arabia; 5 Division of Geriatrics & Nutritional Science, Washington University School of Medicine, St. Louis, USA

**Keywords:** lipin1, DMD, Muscular dystrophy, Dystrophin, Skeletal muscle, Therapeutic target, Membrane integrity, Phosphatidic acid phosphatase

## Abstract

**Background:**

Duchenne muscular dystrophy (DMD) is a progressive and devastating muscle disease, resulting from the absence of dystrophin. This leads to cell membrane instability, susceptibility to contraction-induced muscle damage, subsequent muscle degeneration, and eventually disability and early death of patients. Currently, there is no cure for DMD. Our recent studies identified that lipin1 plays a critical role in maintaining myofiber stability and integrity. However, lipin1 gene expression levels are dramatically reduced in the skeletal muscles of DMD patients and mdx mice.

**Methods:**

To identify whether increased lipin1 expression could prevent dystrophic pathology, we employed unique muscle-specific mdx:lipin1 transgenic (mdx:lipin1^Tg/0^) mice in which lipin1 was restored in the dystrophic muscle of mdx mice, intramuscular gene delivery, as well as cell culture system.

**Results:**

We found that increased lipin1 expression suppressed muscle degeneration and inflammation, reduced fibrosis, strengthened membrane integrity, and resulted in improved muscle contractile and lengthening force, and muscle performance in mdx:lipin1^Tg/0^ compared to mdx mice. To confirm the role of lipin1 in dystrophic muscle, we then administered AAV1-lipin1 via intramuscular injection in mdx mice. Consistently, lipin1 restoration inhibited myofiber necroptosis and lessened muscle degeneration. Using a cell culture system, we further found that differentiated primary mdx myoblasts had elevated expression levels of necroptotic markers and medium creatine kinase (CK), which could be a result of sarcolemmal damage. Most importantly, increased lipin1 expression levels in differentiated myoblasts from mdx:lipin1^Tg/0^ mice substantially inhibited the elevation of necroptotic markers and medium CK levels.

**Conclusions:**

Overall, our data suggest that lipin1 is a promising therapeutic target for the treatment of dystrophic muscles.

**Supplementary Information:**

The online version contains supplementary material available at 10.1186/s12967-024-05494-z.

## Background

Duchenne muscular dystrophy (DMD) is a severe and progressive disease that affects approximately 1 in 3500 male births worldwide [1–3]. Symptoms manifest as impaired movement at age 3–5 years, patients often require a wheelchair before 12 years of age [4, 5], and die around age 30 [6, 7]. DMD is caused by mutations in the gene coding for dystrophin, a key protein for maintaining the structural integrity of the muscle cell membrane [8]. Loss of dystrophin leads to membrane damage during muscle contraction, as well as repeated cycles of degeneration and regeneration of muscle fibers. Ultimately, the regenerative capacity of muscle becomes exhausted, resulting in progressive muscle weakness and early mortality in affected teenagers and young adults.

Currently, there is no cure for this disease. Glucocorticoid treatment is effective for many inflammatory disorders; however, long-term treatment is associated with significant adverse effects including weight gain, increased risk for infection, gastric ulcers, hypertension, diabetes, etc. Duvyzat, a histone deacetylase (HDAC) inhibitor, has been shown to reduce inflammation and improve muscle strength. However, Duvyzat has side effects including diarrhea, abdominal pain, bleeding, nausea, obesity, and fever. The primary strategy for the treatment of DMD is to reverse membrane instability by increasing dystrophin levels, however, this represents a major obstacle in that adeno-associated virus (AAV) has a maximum packaging capacity of 4.7 kb, and dystrophin is encoded by an approximately 11.4 kb cDNA. Therefore, current viral vectors are unable to carry and effectively deliver such a large gene. Elevidys micro-dystrophin gene therapy has recently been approved by FDA for the treatment of DMD, but micro-dystrophin and mini-dystrophin use highly truncated forms of dystrophin, which may prevent them from offering full protection [9–11]. Amondys 45 Exondys 51, Viltepso, and Vyondys 53 are FDA-approved exon skipping therapies to put the mRNA back in-frame [12–14]. However, these approaches are only applicable to a subset of DMD patients with a targeted mutation. Therefore, there is an urgent need to identify new potential therapeutic targets for the treatment of DMD.

Lipin1 is an intracellular protein that plays an important role in maintaining membrane integrity [15, 16]. Lipin1 is a phosphatidic acid (PA) phosphatase (PAP) that catalyzes the conversion of PA to diacylglycerol (DAG), a critical step in the synthesis of glycerophospholipids [17, 18]. Patients with lipin1 deficiency exhibit severe episodic and recurrent rhabdomyolysis in early childhood [19], and mice lacking lipin1 in skeletal muscle exhibit progressive skeletal myopathy [20–23]. Our previous studies revealed that lipin1 is critical for the maintenance of membrane integrity and lipin1 deficiency alone leads to compromised muscle membrane integrity [15]. Loss of membrane integrity leads to sarcolemmal damage and muscle degeneration [15]. Lipin1 plays a complementary role to dystrophin in myofiber stability [16], and knockout of lipin1 in dystrophic muscle produced a more severe phenotype characterized by exacerbated sarcolemmal damage, and increased necroptosis and fibrosis in dystrophin:lipin1 double knockout mice compared to mdx mice [16, 24]. Complementation of lipin1 reversed the elevation of necroptotic markers in differentiated primary lipin1-deficient myoblasts [16]. Despite the critical role of lipin1 in maintaining myofiber stability, we identified that lipin1 gene expression levels are dramatically reduced in dystrophic muscles of DMD patients and mdx mouse models [16].

In this study, we hypothesized that increased lipin1 expression levels could ameliorate the dystrophic phenotype in skeletal muscle of mdx mice. To test our hypothesis, a novel muscle-specific mdx:lipin1 transgenic mouse model and a gene delivery approach via intramuscular injection were used to examine the effects of lipin1 restoration on preventing the pathophysiology of mdx mice. We showed that lipin1 restoration in dystrophic muscle leads to strengthened membrane integrity and lessened muscle fiber degeneration, which resulted in improved muscle force and performance suggesting that lipin1 could be a potential therapeutic target for the treatment of DMD.

## Methods

### Ethical approval

All animal experiments were performed in accordance with the relevant guidelines and regulations approved by the Animal Care and Use Committee of Wright State University and approval was obtained for all experiments performed in this study (#1218 and #1206). For tissue collection, mice were euthanized via isoflurane overdose followed by cervical dislocation prior to tissue harvest. For primary myoblast isolation, three-to-five-day old pups were euthanized by narcotization with isoflurane followed by rapid decapitation with sharp scissors. All efforts were made to minimize animal suffering.

### Animals

**Fig. 1:**
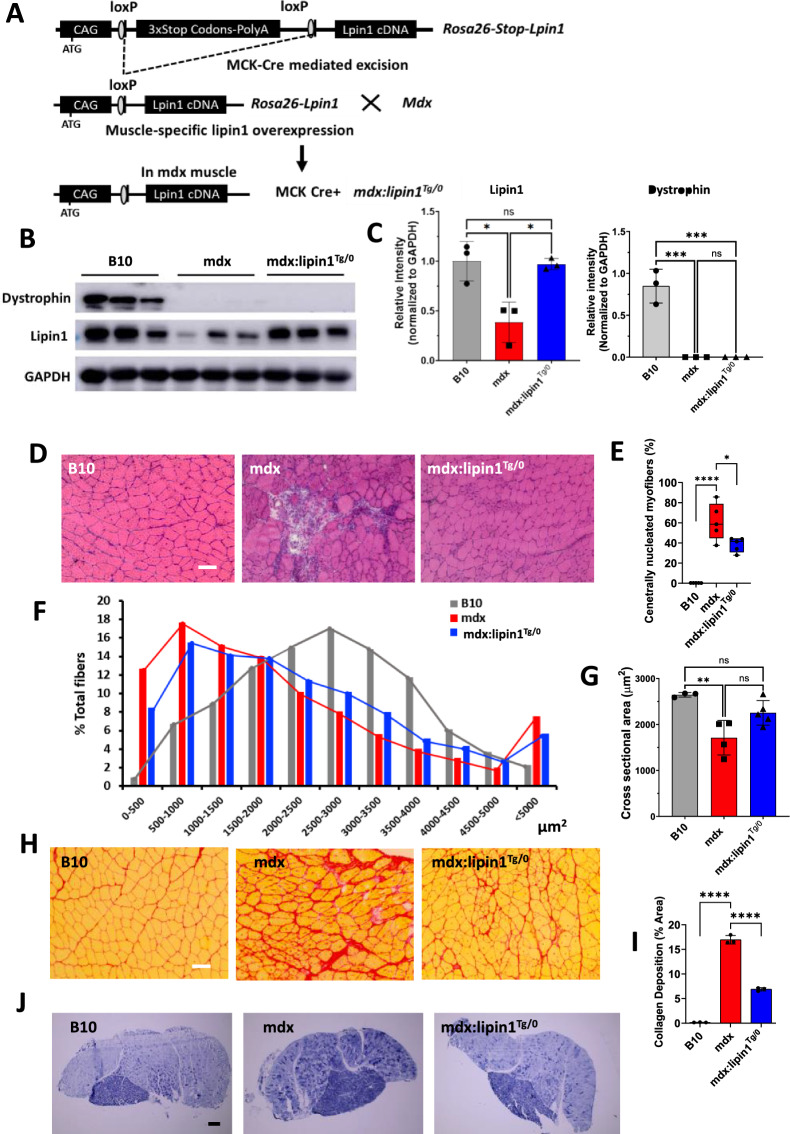
Overexpression of lipin1 attenuated dystrophic histology in gastrocnemius of muscle-specific mdx:lipin1^Tg/0^ mice. **A** Schematic strategy for generating mdx:lipin1^Tg/0^ mice. **B** Representative immunoblot and **C** quantitative densitometry analysis of dystrophin and lipin1 protein in the gastrocnemius muscle of B10, mdx, and mdx:lipin1^Tg/0^ mice. **D** Representative H&E staining, **E** quantification analysis of centrally nucleated myofibers, **F** cross-sectional areas of muscle fibers, **G** average cross-sectional area, **H** picrosirius red staining, and **I** quantification analysis of collagen deposition of the gastrocnemius muscle in B10, mdx, and mdx:lipin1^Tg/0^ mice (Scale bars = 100um). *p < 0.05; ****p < 0.0001. **J** NADH staining of the gastrocnemius muscle in B10, mdx, and mdx:lipin1^Tg/0^ mice (Scale bars = 500um). n = 3–5 mice per group

C57BL/10ScSnJ (B10, #000476) wild-type (WT) and mdx (#001801) mice, purchased from Jackson Laboratories (Bar Harbor, ME, USA). Skeletal muscle-specific lipin1 transgenic (Rosa26-lipin1^KI^) mice were generated by Dr. Brian Finck at Washington University in St. Louis by knocking in a cassette containing a mouse lipin1 cDNA into the ROSA26 locus by using TALENS. This cassette contains, in sequence, the constitutive chicken α-actin promoter, 3 stop codons flanked by LoxP sites, and the cDNA for mouse lipin1. ES cells with this allele knocked in were injected into developing mouse embryos. Resulting Rosa26-stop-Lpin1 founder mice were bred with mice expressing Cre recombinase driven by muscle creatine kinase (MCK) gene promoter, thus removing the stop codons from the cassette in skeletal muscle DNA and allow for myocyte specific overexpression of lipin1. We further crossed Rosa26-lipin1^KI^ with mdx mice and generated hemizygous mdx:lipin1 transgenic mice with one copy of lipin1 transgene in skeletal muscle of mdx mice (Fig. 1A). We also crossed hemizygous mdx:lipin1 transgenic mice and generated mdx mice harboring lipin1 transgene but do not express MCK-Cre, which share similar gastrocnemius muscle morphology, fibrosis, inflammation markers, cell death markers and membrane integrity with mdx mice to rule out any potential effects introduced by genetic backgrounds. Therefore, mdx mice were used as controls. As DMD occurs primarily in males [5], male mice were used in the current study. These mice had free access to drinking water and regular chow, unless otherwise noted.

### Western blotting

Muscle tissues were lysed in RIPA buffer containing 10 mM Tris–HCl (pH 7.4), 30 mM NaCl, 1 mM EDTA and 1% Nonidet P-40, supplemented with proteinase inhibitors and phosphatase inhibitors before use. Protein concentration was determined for each sample and equal amounts of proteins were used, boiled at 95 °C for 5 min in 1% of SDS sample buffer and separated by 7.5–15% SDS-PAGE. Thereafter, proteins were transferred to polyvinylidene difluoride membranes (Millipore) using a Mini Trans-Blot Cell System (Bio-Rad). The membrane was blocked with 5% nonfat milk (Cell Signaling Technologies, 9999) for 1 h, and incubated with the primary antibodies in 5% BSA (Thermo Fisher Scientific, BP9704) in TBST overnight at 4 °C. After probing with secondary antibodies for 1 h at 25 °C, protein bands were detected by using Amersham Imager 600 (GE Healthcare Life Sciences). Gapdh (Cell Signaling Technologies, 2118, 1:5000) antibody was used as a loading control. The densitometry values were normalized by the corresponding loading control densitometry values. Antibodies used include lipin1 (Cell Signaling Technology, 14906, 1:1000), RIPK1 (Cell Signaling Technology, 3493, 1:1000), RIPK3 (Cell Signaling Technology, 95702, 1:1000), MLKL (Cell Signaling Technology, 37705, 1:1000), Bid (Cell Signaling Technology, 2002, 1:1000), Bax (Cell Signaling Technology, 2772, 1:1000), Bak (Cell Signaling Technology, 12105, 1:1000), cleaved caspase 3 (c.cas 3, Cell Signaling Technology, 9661, 1:1000), NFkB (Cell Signaling Technology, 8242, 1:1000), phospho-NFkB (Cell Signaling Technology, 3033, 1:1000), along with goat anti-Rabbit IgG-HRP (Cell Signaling Technology, 7074, 1:2000).

### Immunofluorescence, microscopy, and image processing

The gastrocnemius muscles were frozen and sectioned at 10 µm using a cryostat machine. Slides were stored at − 20 °C. For staining, muscle sections were air dried for 1 h at room temperature. The muscle sections were then hydrated with PBST, followed by blocking with 5% goat serum in PBST. To detect the restoration of lipin1 in AAV1-lipin1 injected gastrocnemius muscle, sections were incubated with antibodies against anti-HA (Abcam, ab18181, 3 µg/ml) and laminin (Abcam, ab11575, 1:200) at 4 °C overnight, and subsequently with an Alexa Fluor 488- or Alexa Fluor 555-conjugated secondary antibody (Thermo Fisher Scientific, A-21411, 1:250) for 1 h in the dark at room temperature. To detect Evans blue dye (EBD) staining, sections were incubated with antibodies against laminin (Abcam, ab11575, 1:500) for 1 h at 37 °C before incubating with secondary antibodies. Images were obtained using an inverted microscope (Olympus, IX70) equipped with a DFC7000T camera (Leica Microsystems, Wetzlar, Germany). Indicated images were quantified by CellProfiler software.

### Generation of adeno-associated viruses (AAV) carrying lipin1 construct

AAV vectors were produced by the Penn Vector Core at the University of Pennsylvania (Philadelphia, PA). To generate AAV virus carrying lpin1 gene, the cDNA encoding for HA-tagged lipin1-beta were inserted into pAAV.CMV.PI.EGFP.WPRE.bGH vector provided by Penn vector Core. pAAV.CMV.PI.EGFP.WPRE.bGH (#105530) were used a controls (Addgene). Plasmids expressing lipin1 or GFP were packaged within AAV1 capsids.

Two-month old mdx mice were injected with HA tagged AAV1-lipin1, AAV1-GFP or saline into their gastrocnemius muscles via intramuscular injections. In each mdx mouse, one leg was injected with AAV-lipin1 (5 × 10^10^ viral genome particles (vg)/mouse), and the contralateral leg with AAV-GFP at same titer or saline. Four-week post injections, mice were sacrificed and gastrocnemius muscles were harvested.

### Evans blue dye (EBD) assay

Mice were injected with EBD (10 mg/ml stock in sterile saline, 0.1 ml/10 g body weight) i.p. and euthanized 24 h later. The skeletal muscles were dissected and snap-frozen in isopentane cooled optimal cutting temperature (OCT) embedding media (Tissue-Tek, Sakura-Americas). Frozen OCT blocks were cryo-sectioned at 10 μm thickness and stained with laminin antibody before being analyzed by fluorescence microscopy.

### Primary myoblasts isolation and culture conditions

Primary myoblasts isolation has been described in our previous studies [21, 24, 25]. Briefly, primary myoblasts were isolated from 3–5-day-old pups of *B10, mdx and Mdx:lipin1*^*Tg/0*^ mice. Hind limb muscles were dissected and minced into pieces. Cells were dissociated (Roche) for 1 h at 37 °C. The slurry was passed through a 70 μm nylon mesh filter (Fisher Scientific, 22363548) and centrifuged for 5 min at 350×*g*. The pellet was resuspended in 10 ml of F10 media containing 20% FBS, 100 U/ml Pen/Strep (GIBCO), and 5 ng/ml bFGF (PeproTech). Same number of primary myoblasts from B10, mdx and mdx:lipin1^Tg/0^ mice were plated in collagen-coated culture plates with an F-10-based primary myoblast growth medium (Ham's F-10 nutrient mixture containing 20% fetal calf serum and 2.5 ng/ml basic fibroblast growth factor), streptomycin, and penicillin and incubated at 37 °C supplied with 5% CO_2_. Primary myoblasts were purified by pre-plating 3 times. The differentiated myotubes were then harvested for protein analysis.

### Body composition analysis

Body composition of mice (total body fat, lean mass) was determined using the whole body quantitative magnetic resonance analyzer EchoMRI-500™ system (EchoMRI LLC, Houston TX) to assess fat and lean mass.

### Contractile force measurement

For muscle force measurements, mice were anesthetized with isoflurane using a low-flow anesthesia system (SS-01, Kent Scientific, Torrington, CT), as described previously [15, 26, 27]. Body temperature was maintained at ~ 35 ℃ using a heat lamp and temperature probe. The plantar flexor muscles (including the lateral and medial gastrocnemius, plantaris, and soleus muscles) and sciatic nerve were exposed by removing the surrounding skin. During surgery, the muscles were bathed in physiological saline to prevent them from drying out. Mice were then transferred to a custom 3D-printed platform filled with Sylgard and mounted to a micromanipulator (XR25/M, Thorlabs, Newton, NJ). The leg was stabilized to prevent movement. The distal end of the plantar flexor muscles (Achilles’ tendon) was attached to the lever of the force transducer (300D-305C dual-mode muscle lever, Aurora Scientific, Ontario, Canada) using 5.0 silk suture and a modified Miller’s knot. Optimal length was obtained by measuring the maximum twitch force while lengthening the muscle using the micromanipulator.

Force-frequency relationships were determined under isometric conditions in response to stimulation of the sciatic nerve using platinum electrodes. Force was measured at each frequency in response to 10–15 stimuli.

Eccentric (lengthening) contraction experiments were achieved using direct-muscle stimulation via platinum electrodes to eliminate any possible effects from altered motor nerve function or neuromuscular transmission. For muscle-stimulated contractions, neuromuscular transmission was blocked using ~ 150 µl of 0.5 mg/ml α-bungarotoxin (α-BTX) injected into the gastrocnemius muscles. After injection of α-BTX, neuromuscular transmission was confirmed to be blocked by ensuring the twitch force was ≤ 25% of maximum nerve-stimulated twitch. Eccentric contractions were measured during a train of 50 stimuli at 100 Hz. Isometric force was measured during the first 200 ms. The contracting muscle was then lengthened for + 0.25 mm over the next 100 ms and then held at + 0.25 mm length for the last 200 ms of stimulation. Following the stimulation, the plantar flexor muscle was returned to optimal length over 100 ms. This protocol was repeated 15 times. The steady-state isometric force during the first 200 ms of each eccentric contraction protocol was measured to assess the decline in force generation during the repeated eccentric contractions.

Stimulus amplitude and the pulse width were ≤ 5 V at 1 ms and ≤ 50 V at 1.5 ms for nerve-stimulated and muscle-stimulated contractions, respectively. A Dagan S-900 Stimulator and S910 Stimulus Isolation Unit were used for stimulation. Muscle force was recorded and digitized using pClamp10 software (Molecular Devices, San Jose, CA).

### Grip strength test and downhill treadmill test

Grip strength test was measured with 2-limbs or all four limbs by a Grip Strength Test Meter (Bioseb, Chaville, France). The final result was calculated using the average of three readings taken at 1–2 min intervals [28, 29]. The body weight of each individual mouse was determined at the end of the experiment to normalize the mean grip strength force to body weight.

Forced treadmill running was utilized to evaluate systemic muscle function. Briefly, mice were initially acclimated to the treadmill (IITC Model 800, IITC Life Science, California) for 30 min on 3 consecutive days (first day at 5 m/min; second and third day at 10 m/min). On the fourth day, mice were placed on a treadmill to run with a downward inclination of 15°, at 5 m/min for 5 min, 10 m/min for 20 min, 15 m/min until exhaustion. The mouse was encouraged to run by using a mild electric shock grid at the end of the treadmill (0.2 mA, pulse 200 ms, 1 Hz). The experimental mouse was considered to be exhausted after its refusal to remain on the treadmill belt for more than 5 s. The time ran at all speeds was recorded until exhaustion.

### Statistical analysis

Quantification analysis of Picrosirius red and EBD staining were used pipelines generated in our lab [30]. For immunostaining and Western blot, statistical analysis was performed with one-way analysis of variance (ANOVA) followed by Bonferroni’s multiple comparison test to determine significant changes between groups using GraphPad Prism 9 (GraphPad Software, version 9.4.0). Data are provided as the mean ± SD number (*n*) of independent experiments.

Force-frequency data were fit with the following Boltzmann equation,

$$=\frac{{F}_{min}-{F}_{max}}{1+{e}^{\left(\frac{{x-freq}_{o.5}}{k}\right)}}+{F}_{max}$$

where,$x=$ frequency of stimulation, $y=$ force, ${F}_{min}=$ minimum force, ${F}_{max}=$ maximum force, ${freq}_{0.5}=$ frequency giving half-max force, and $k=$ slope factor. Data were analyzed with OriginPro 2022 (OriginLab Corp.) and reported as mean ± SD. Multiple means were compared with a one-way ANOVA, post hoc Bonferroni. For all tests, groups were considered statistically different at α = 0.05.

## Results

### Increased lipin1 expression ameliorated the dystrophic phenotype in mdx:lipin1^Tg/0^ mice

Lipin1 transgenic mice were generated by knocking in a lipin1beta cDNA into the ROSA26 locus and crossing founder mice with mice expressing Cre under the control of the muscle creatine kinase (MCK) promoter (Fig. 1A). To evaluate the effect of increasing lipin1 expression levels on dystrophic pathology, we crossed muscle-specific lipin1 transgenic mice with mdx mice to generate muscle-specific mdx:lipin1 transgenic (mdx:lipin1^Tg^) mice. Western blotting analysis showed that the protein expression levels of lipin1 in gastrocnemius of hemizygous mdx:lipin1^Tg/0^ mice were restored back to physiologic levels similar as in B10 WT mice (Fig. 1B, C). Therefore, the mdx:lipin1^Tg/0^ mice used in this study show the effects of restoring lipin1 expression in mdx muscle to normal physiological levels. Results are shown compared to mdx and B10 mice.

We compared the morphology of gastrocnemius muscle sections in 5-month-old B10 WT, mdx:lipin1^Tg/0^ and mdx mice by H&E staining (Fig. 1D). The gastrocnemius of mdx mice revealed exaggerated inflammatory infiltration as well as 61 ± 18% of centrally-located nuclei compared to the age-matched B10 WT mice (Fig. 1E). Restoration of lipin1 suppressed inflammatory cell infiltration and reduced the percentage of centrally nucleated myofibers to 38 ± 7% in mdx:lipin1^Tg/0^ compared to mdx mice, indicating less muscle degeneration (Fig. 1E). Since mdx muscle has a high heterogeneity of myofiber size due to continuous cycles of muscle degeneration and regeneration, we also measured the cross-sectional areas of gastrocnemius muscle fibers in these three groups. The average of myofiber cross-sectional area in B10 mice was 2643 μm^2^ (Fig. 1F, G). Restoration of lipin1 in mdx: lipin1^Tg/0^ mice caused an increased mean myofiber cross-sectional area of 2252 μm^2^ compared to 1710 μm^2^ in mdx controls (Fig. 1F, G). The increase in myofiber CSA in mdx:lipin1^Tg/0^ mice may be due to reduced muscle degeneration and the maturation of regenerating myofibers.

Fibrosis is a hallmark of DMD, leading to muscle wasting and impaired muscle functions. We evaluated muscle fibrosis by Picrosirius red staining in B10, mdx, and mdx:lipin1^Tg/0^ mice (Fig. 1H, I). Picrosirius red staining revealed a significantly decreased collagen deposition in gastrocnemius of 5-month-old male mdx:lipin1^Tg/0^ mice compared to age-matched mdx mice (17% vs. 7% of total area, *p* < *0.0001*), suggesting a prevention of fibrosis in mdx:lipin1^Tg/0^ mice.

We also performed Nicotinamide adenine dinucleotide (NADH) staining on sections of gastrocnemius of B10, mdx, and mdx:lipin1^Tg/0^ mice to determine the oxidative potential of myofibers (Fig. 1J). The mdx muscles had more dark and intermediate staining showing an increased population of oxidative fibers whereas B10 WT gastrocnemius muscles showed a mosaic pattern of darkly stained oxidative and lightly stained glycolytic fibers (Fig. 1J). The abnormal fiber-type distribution seen in mdx muscles was improved in mdx:lipin1^Tg/0^ muscle. Overall, we observed that restoration of lipin1 expression levels in mdx muscle reduced the number of centrally-nucleated myofibers and fibrosis.

### Restoration of lipin1 in mdx:lipin1^Tg/0^ mice prevented the inflammatory pathology

**Fig. 2:**
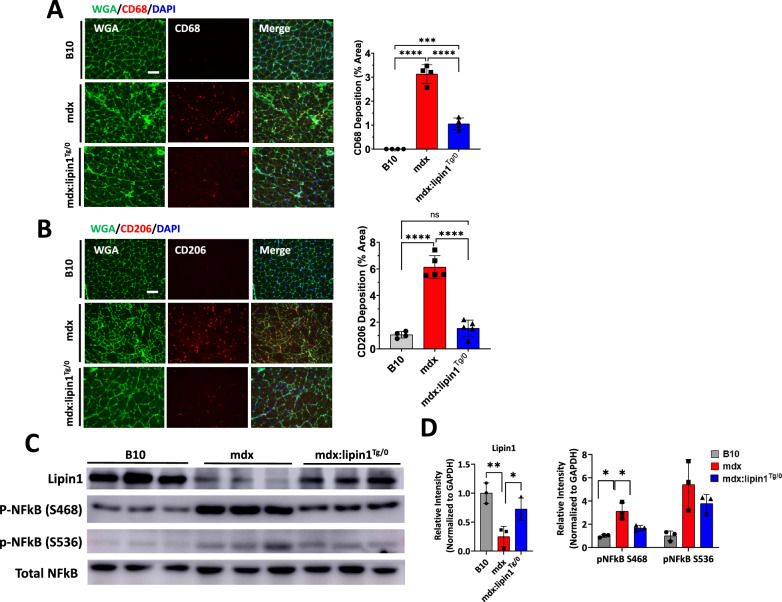
Restoration of lipin1 suppressed inflammation in dystrophic muscle. Representative immunostaining and quantification analysis of **A** CD68 and **B** CD206 expression (red) in gastrocnemius muscle cross-sections from B10, mdx, and mdx:lipin1^Tg/0^ mice. Wheat germ agglutinin (WGA, green) was used to visualize muscle fibers (Scale bars = 100um). n = 3–5 mice per group. *p < 0.05; ***p < 0.001; ****p < 0.0001. **C** Representative immunoblots and **D** quantitative analysis of inflammation (p-NFkB and NFkB) markers in indicated mice. n = 3 mice/group. *p < 0.05; **p < 0.01; ***p < 0.001

Inflammatory macrophages have been shown to play a central role in muscle pathology in DMD patients [31] and mdx mice [32]. CD68^+^ macrophages play a vital role in phagocytosing debris after muscle injury, whereas, CD206^+^ macrophages are involved in fibrosis progression [33, 34]. The abundance and distribution of macrophages in B10, mdx, and mdx:lipin1^Tg/0^ muscle were detected by immunostaining with antibodies to detect CD68 and CD206, respectively (Fig. 2A, B). Wheat germ agglutinin (WGA) staining was used to identify the myofiber border. The surface area per cross-sectional area of CD68^+^ macrophage was increased to 3.1% (*p* < *0.0001*) in mdx compared to WT muscle, but was reduced to 1.1% (*p* < *0.0001*) in mdx:lipin1^Tg/0^ muscle (Fig. 2A). Moreover, CD206^+^ M2 macrophage was increased to 6.1% (*p* < *0.0001*) in mdx compared to 1.1% in WT muscle, but was reduced to 1.5% (*p* < *0.0001*) in mdx:lipin1^Tg/0^ muscle (Fig. 2B).

We further evaluated the effects of restoration of lipin1 in dystrophic muscle inflammation and degeneration by Western blot analysis (Fig. 2C, D). Firstly, Western blot analysis of lipin1 in gastrocnemius of mdx:lipin1^Tg/0^ mice showed that the lipin1 protein expression levels were restored similarly as in WT controls. The NF-κB is a key transcription factor of M1 macrophages and is a master regulator of inflammatory response [35]. Phosphorylation at 536 and 468 serine residues is thought to be required for the activation and nuclear translocation of NF-κB [36]. To identify the effect of lipin1 restoration in muscle inflammation, we measured the activation of NF-kB in gastrocnemius of 6-month-old mdx:lipin1^Tg/0^ mice and their mdx controls (Fig. 2C, D). We observed increased phosphorylation of NF-kB at serine 468 residues (212%) in mdx compared to B10 (*p* < *0.05*), indicating activation of inflammation in mdx muscle. Mdx:lipin1^Tg/0^ muscles had substantially reduced inflammation indicated by reduced expression level of phosphor-NF-kB at serine 468 residues by 147% compared to mdx muscles (*p* < *0.05*). These results indicate that restoration of lipin1 reduced inflammation in mdx mice.

### Restoration of lipin1 slowed down the degeneration-regeneration cycles in dystrophic muscle

**Fig. 3:**
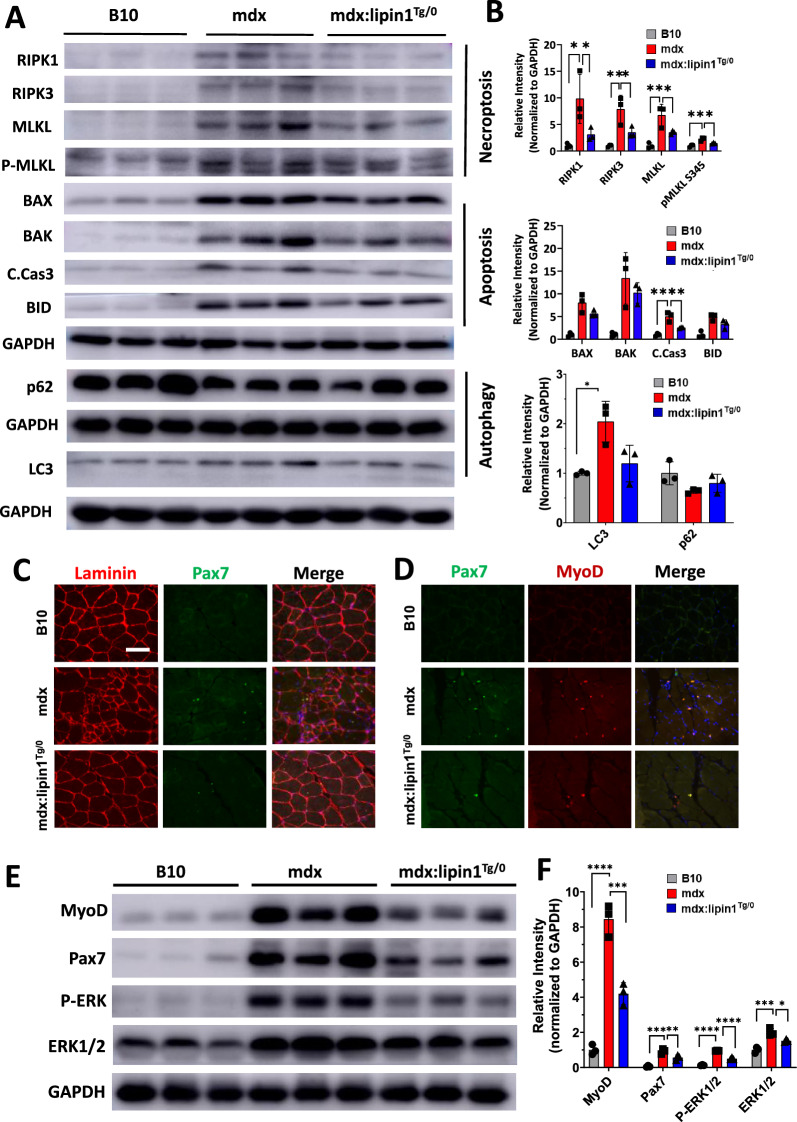
Restoration of lipin1 in dystrophic muscle slowed down the degeneration-regeneration cycle. **A** Representative immunoblots and **B** quantitative analysis of necroptotic (RIPK1, RIPK3, and MLKL), apoptotic (BAX, BAK, BID, and cleaved Cas 3 (C.Cas 3)), and autophagy (p62 and LC3) markers in indicated mice. n = 3 mice/group. *p < 0.05; **p < 0.01; ***p < 0.001. **C** Immunostaining of pax7 (green) and laminin (red), and **D** pax7 (green) and myoD (red) in gastrocnemius of B10, mdx and mdx:lipin1^Tg/0^ mice. **E** Western blot and **F** quantification analysis of indicated markers in gastrocnemius of B10, mdx and mdx:lipin1^Tg/0^ mice (n = 3 mice/group; *p < 0.05; **p < 0.01; ***p < 0.001; ****p < 0.0001)

Necroptosis, a form of regulated necrotic cell death, is mediated by receptor-interacting serine/threonine-protein kinase 1 (RIPK1), RIPK3, and mixed-lineage kinase-domain-like pseudokinase (MLKL) which contribute to muscle degeneration. In particular, RIPK3 is thought to be the major driver of limb muscle degeneration at the onset of DMD pathogenesis [37]. As shown in Fig. 3A and B, We found that restoration of lipin1 substantially reduced the expression levels of RIPK1, RIPK3, MLKL, and phospho-MLKL (p-MLKL) by 68%, 57%, 48%, and 36% respectively in mdx:lipin1^Tg/0^ compared to mdx mice. Apoptosis has also been suggested to contribute to the disease progression of DMD [38], therefore, in this study, we also measured the apoptotic markers. We found that the apoptotic executioner, cleaved caspase 3, was reduced by 50% in gastrocnemius of mdx:lipin1^Tg/0^ compared to mdx mice. Moreover, autophagy has been shown to be impaired in skeletal muscles from DMD patients or mdx mice [39]. We also measured p62 and LC3 levels in gastrocnemius of B10, mdx, and mdx:lipin1^Tg/0^ mice. We found that LC3-I was increased in mdx gastrocnemius muscle compared to B10 WT control suggesting a defective autophagy in skeletal muscle of dystrophic muscle. This is consistent with previous studies [39]. Although we did not detect a significant difference in p62 and LC3 between mdx and mdx: lipin1^Tg/0^ muscle, increasing lipin1 expression seems to improve the autophagy defects in mdx muscle (Fig. 3A, B). Overall, these results indicate that restoration of lipin1 reduced muscle degeneration in mdx mice.

To evaluate the muscle regeneration in mdx:lipin1^Tg/0^ mice, we performed co-staining of pax7 and laminin, as well as pax7 and myoD (Fig. 3C, D). We found that both pax7 and myoD were detected in gastrocnemius of mdx and mdx:lipin1^Tg/0^ mice. Most of the pax7-positive cells were also myoD-positive. Overall, mdx:lipin1^Tg/0^ mice had less pax7 and myoD expression compared to mdx muscle. We also measured the protein expression levels of pax7 and myoD (Fig. 3E, F). Consistently, the protein expression levels of pax7 were highly elevated in mdx muscle, but were reduced in gastrocnemius of mdx:lipin1^Tg/0^ mice. In addition, our previous study showed that ERK promotes cell proliferation and is activated during muscle regeneration [25]. Indeed, we found that ERK was highly activated in mdx muscle, which is consistent with previous studies [40]. Increasing lipin1 expression levels in mdx:lipin1^Tg/0^ muscle had reduced ERK activation Fig. 3E, F). The elevated pax7, myoD, and ERK in mdx muscle were likely due to activated muscle regeneration caused by continuous muscle degeneration. In contrast, mdx:lipin1^Tg/0^ mice had much less muscle damage, and therefore manifested lower regeneration compared to mdx mice. Overall, we found that restoration of lipin1 slowed down the degeneration-regeneration cycles in dystrophic muscle.

### Restoration of lipin1 levels in mdx:lipin1^Tg/0^ mice improved muscle membrane integrity and function

**Fig. 4:**
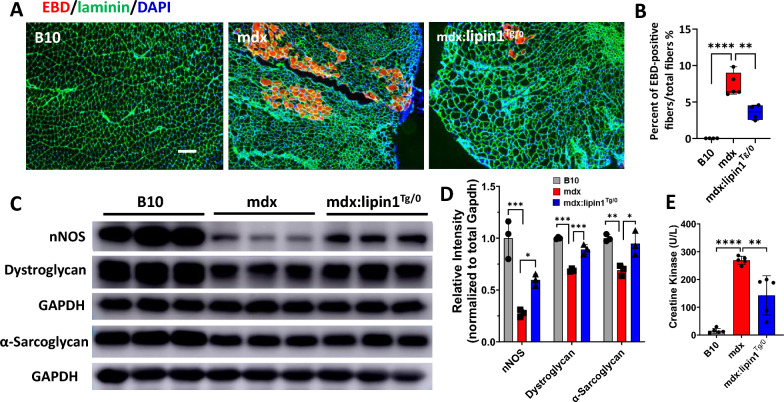
Restoration of lipin1 in dystrophic gastrocnemius improved sarcolemmal integrity. **A** EBD uptake (red) of gastrocnemius muscle sections from B10, mdx, and mdx:lipin1^Tg/0^ mice. Laminin (green) was used to visualize muscle fibers. Scar bar = 200μm. **B** Quantification analysis of EBD-positive muscle fiber expressed as the percentage of the total number of muscle fibers in each mouse. n = 4–5 mice/group. **p < 0.01, ****p < 0.0001. **C** Protein expression levels and **D** quantification analysis of dystroglycan, α-sarcoglycan, and nNOS in gastrocnemius of B10, mdx, and mdx:lipin1^Tg/0^ mice. n = 3 mice/group. *p < 0.05; **p < 0.01, ***p < 0.001. **E** Serum creatine kinase levels in indicated mice. n = 5 mice/group. **p < 0.01; ****p < 0.0001

Loss of dystrophin in mdx mice results in membrane disruption and muscle damage during muscle contraction and relaxation cycles. Our recent studies showed that lipin1 plays an important role in the maintenance of membrane integrity [15, 24]. To determine whether increasing lipin1 levels ameliorate muscle membrane disruption in mdx mice, we compared muscle membrane permeability in mdx:lipin1^Tg/0^ with mdx mice by EBD uptake. B10 mice injected with EBD were used as negative controls. As shown in Fig. 4A, B, we did not detect any EBD^+^ myofiber in B10 muscle, but gastrocnemius of mdx mice had 7.4% EBD^+^ myofibers (*p* < *0.0001*) suggesting these myofibers were undergoing membrane disruptions and muscle damage. Most importantly, restoration of lipin1 in mdx:lipin1^Tg/0^ mice had substantially reduced EBD-positive fibers to 3.5% (*p* < *0.01*), indicating that lipin1 upregulation reduced the extent of muscle damage in mdx mice which possibly through increasing sarcolemma stability in mdx mice.

To further evaluate the roles of lipin1 in muscle fiber stability, we measured the protein expression levels of some of the dystrophin-associated protein complex (DAPC) in gastrocnemius of B10, mdx, and mdx:lipin1^Tg/0^ mice via Western blot analysis (Fig. 4C, D). Consistent with previous studies [41], dystrophin deficiency led to loss of protein expression levels of α-sarcoglycan, dystroglycan, and nNOS that resulted in sarcolemmal instability. In contrast, these structural molecules were improved in mdx:lipin1^Tg/0^ mice.

Elevated serum levels of creatine kinase (CK) have been generally considered to be an indirect marker of muscle damage. We then measured serum CK levels in B10, mdx and mdx:lipin1^Tg/0^ mice. We found that mdx mice had elevated CK levels and increasing lipin1 expression levels in mdx:lipin1^Tg/0^ mice reduced CK levels (Fig. 4E). Overall, these findings suggest a critical role for lipin1 in maintaining myofiber stability and integrity.

### Lipin1 restoration improved in situ muscle contractile and eccentric force in mdx:lipin1^Tg/0^ mice

**Fig. 5:**
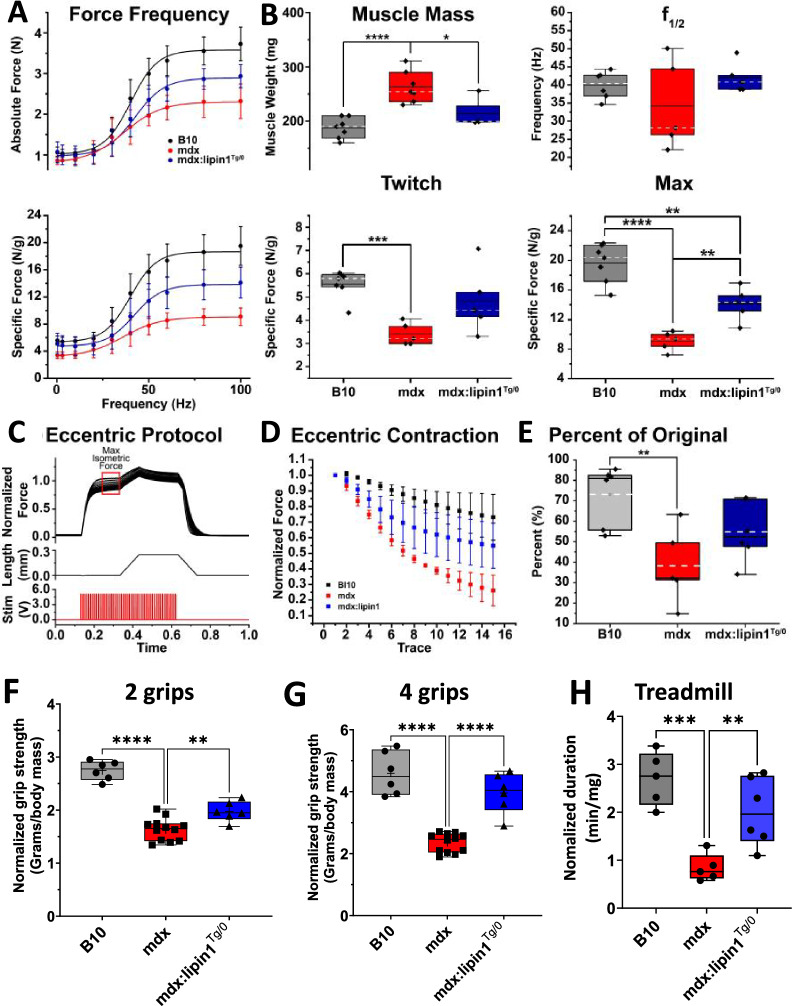
Restoration of lipin1 improved dystrophic muscle force production, grip strength, and running capability. **A** Absolute (*top*) and specific (*bottom*) force-frequency curves for B10 (black curve), mdx (red curve), and mdx:lipin1^Tg/0^ (blue curve) plantar flexor muscles stimulated via the sciatic nerve. **B** Box-and- whisker plots of muscle mass (*top left*) used to obtain specific force, the frequency of half maximal contraction (f1/2 max, *top right*), specific twitch force (*lower left*), and the maximum tetanic force at 100 Hz stimulation (*lower right*). Boxes represent the interquartile range, black line represents the median, and the dashed white line represents the mean of the data. **C** Representative trace of In situ eccentric force protocol. The upper panel shows 15 normalized force traces. Traces were normalized by dividing each trace by the max isometric force value indicated by the red box on the graph. The middle and lower panels show the length and stimulation of the protocol respectively. **D** Scatter plot of eccentric contractions for B10 (black curve), mdx (red curve), and mdx:lipin1^Tg/0^ (blue curve) plantar flexor muscles. Force values were normalized to the first trace to assess the decline in force caused by muscle damage sustained through contractions while the muscle was stretched. **E** Box-and-whisker plots of the percent of the original force to assess loss of force caused by the eccentric force protocol. Values are shown as ± SEM. n = 5–6 mice/group. *p < 0.05; **p < 0.01; ***p < 0.005; ****p < 0.001. **F** Two-grip or **G** four-grip strength test in B10, mdx and mdx:lipin1^Tg/0^ mice. **H** Age and gender-matched 5-month-old WT, mdx, and mdx:lipin1^Tg/0^ mice were subjected to forced downhill running. Muscle performance was measured as total running time to exhaustion normalized to the body weight of mice (n = 5–6 mice/group). **p < 0.01; ***p < 0.001; ****p < 0.0001

To test the functional effects of increasing lipin1 expression in mdx muscle, we first measured the force-frequency relationship of control B10, mdx, and mdx:lipin1^Tg/0^ plantar flexor muscle (medial and lateral gastrocnemius, plantaris, and soleus) under in situ conditions (Fig. 5A, B). The force-frequency relationship was assessed by measuring the average peak muscle force in response to 10–15 stimuli at frequencies ranging from 0.3 to 100 Hz and is shown for the absolute force (N) in the top panel of Fig. 5A. Stimulation at 0.3 Hz triggered twitches, whereas, 100 Hz stimulation induced maximum tetanic contractions. The absolute force generated by B10 muscle was substantially greater than that of mdx muscle (Fig. 5A, *top panel*), particularly at high frequencies of stimulation. Increased hemizygous expression of lipin1 in mdx muscle (mdx:lipin1^Tg/0^) resulted in absolute force that was greater than mdx and less than B10 muscle.

Normalizing the muscle force to account for muscle mass (N/g) increased the differences of mdx and mdx:lipin1^Tg/0^ muscle force compared to B10 (Fig. 5A, *bottom panel*). This occurred because of the characteristic pseudohypertrophy of mdx muscle, which is thought to be due to increased inflammation and fibrosis stemming from increased muscle degeneration in mdx muscle [42, 43]. As a result, the mdx muscle was 40% more massive than control B10 (Fig. 5B, *upper left*, *p* < *0.001*). The added non-muscle fiber mass likely further hinders mdx muscle function. However, increasing lipin1 expression in the mdx muscle appeared to decrease the pseudohypertrophy, as the mass of the mdx:lipin1^Tg/0^ muscle was significantly less than mdx by 19% (*p* < *0.05*) but not significantly different than B10 (*p* < *0.05*) (Fig. 5B, *upper left*). Further suggesting that increased expression of lipin1 decreased the degeneration in mdx muscle which are consistent with the above data showing decreased inflammation and fibrosis in mdx:lipin1^Tg/0^ compared to mdx muscle. Examination of specific force, which is independent of muscle mass and pseudohypertrophy, revealed that the average mdx twitch (Fig. 5B, *bottom left*) and maximum tetanic (Fig. 5B, *bottom right*) forces were significantly lower than that of B10 control (*p* < *0.005)* for twitch and (*p* < *0.0001)* for max tetanic force), consistent with previous reports [24]. Peak mdx:lipin1^Tg/0^ twitch force was between that of mdx and B10 but not significantly different from either. The maximum tetanic force of mdx:lipin1^Tg/0^ muscle was significantly greater than that of mdx (*p* < *0.01*) and significantly less than B10 (*p* < *0.005*). This data indicates that increased expression of lipin1 ameliorates the pseudohypertrophy and improves force generation in mdx muscle.

It has been established that the mdx muscle undergoes greater damage during lengthening (eccentric) contractions compared to control muscle [44, 45]. To measure the resulting loss of contractility during eccentric contractions, we measured in situ plantar flexor force during a repeating 100 Hz, 50 stimulation protocol (Fig. 5C). During each eccentric protocol, the maximum steady state isometric force at 100 Hz was measured before the plantar flexor muscle was lengthened by 0.25 mm over 100 ms and then held at + 0.25 mm length for 200 ms, all while maintaining 100 Hz stimulation. After the + 0.25 holding phase, the stimulations were stopped and the muscle was returned to optimal length. This eccentric protocol was repeated 15 times. The maximum steady-state isometric force was measured for each eccentric protocol to determine the decline in force production during the repeated lengthening protocols. The fraction of the initial force during each of the 15 eccentric protocols for B10, mdx:lipin1^Tg/0^, and mdx muscle is shown in Fig. 5D. B10 muscle retained 73.1 ± 0.6% of its initial force during the eccentric protocols, which was significantly greater than the 26.1 ± 0.1% retained by mdx muscle (*p* < *0.01*, Fig. 5E). The 54.8 ± 0.1% of force retained by mdx:lipin1^Tg/0^ muscle force was not significantly different than B10 or mdx, suggesting that increased lipin1 expression in mdx muscle decreases the damage during lengthening contractions.

### Lipin1 restoration improved the grip strength and running capability in mdx:lipin1^Tg/0^ mice

To further identify the functional role of lipin1 restoration in mdx mice, we measured the muscle performance of B10, mdx, and mdx:lipin1^Tg/0^ mice via grip strength test (Fig. 5F, G). Grip strength was measured in grams by a grip-strength meter on conscious mice, and normalized to their body weight. We found that mdx exhibited decreased forelimb and four-limb grip strength, but restoration of lipin1 significantly increased the forelimb (*p* < *0.0001*) and four-limb (*p* < *0.01*) grip strength (Fig. 5F, G).

We also assessed the running capability of hemizygous mdx:lipin1^Tg/0^ compared to mdx controls via a downhill treadmill test (Fig. 5H). B10 WT mice were used as controls. We found that the mdx mice had reduced running endurance compared to B10 WT mice (*p* < *0.0001*) and that restoration of lipin1 in mdx:lipin1^Tg/0^ increased time-to-fatigue in treadmill test (42 min vs. 17 min), as compared to mdx controls (*p* < *0.01*).

### Restoration of lipin1 corrected reduced fat in mdx mice and did not cause lipid deposition in dystrophic muscle

**Fig. 6:**
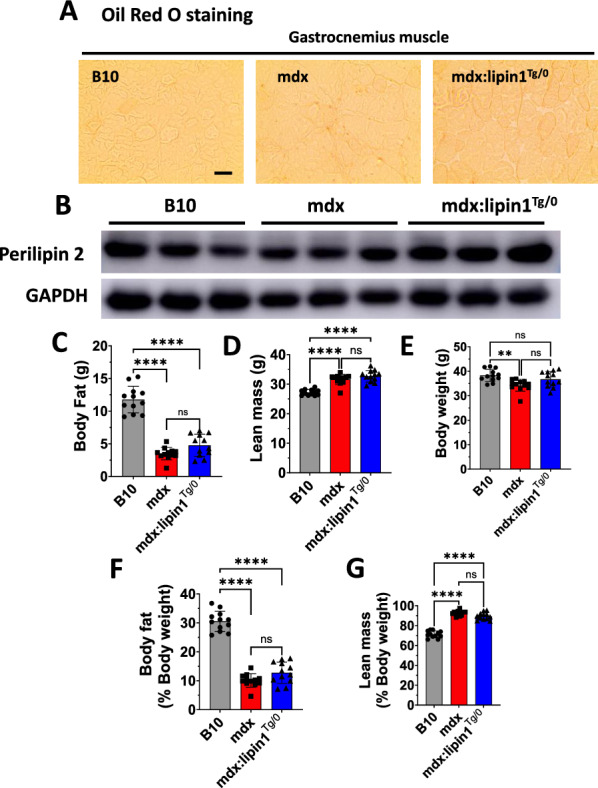
Increasing lipin1 expression levels in dystrophic muscle did not result in obvious side effects. **A** Oil Red O staining of gastrocnemius of 6-month-old WT, mdx, and mdx:lipin1^Tg/0^ mice (scale bar = 100μm; n = 3 mice/group). **B** Western blot analysis of perilipin 2 in gastrocnemius of B10, mdx and mdx:lipin1^Tg/0^ mice (n = 3 mice/group). Body composition including **C** body fat, **D** lean body mass, **E** body weight, **F** body fat to body weight ratios, and **G** lean body mass to body weight ratios in 6–7-month-old of B10, mdx, and mdx:lipin1^Tg/0^ mice was determined using EchoMRI (n = 12 mice/group). Ns, no significant significance; *p < 0.05; ***p < 0.001; ****p < 0.0001

The potential adverse effects of lipin1 restoration in mdx mice was also evaluated. Since lipin1 acts as a phosphatidic acid phosphatase and is involved in lipid synthesis, we examined whether restoration of lipin1 in dystrophic muscle may lead to lipid deposition in gastrocnemius muscle of mdx:lipin1^Tg/0^ mice. We performed Oil Red O staining and did not detect intramuscular lipid accumulation in gastrocnemius of 6-month-old of B10, mdx, and mdx:lipin1^Tg/0^ mice (Fig. 6A). Perilipin 2 is the most abundant lipid droplet coating protein in skeletal muscle. We also measured the protein expression levels of perilipin 2 in gastrocnemius of these groups (Fig. 6B). We did not detect altered perilipin 2 expression in mdx:lipin1^Tg/0^ compared to mdx mice suggesting that lipin1 restoration will not lead to the risk for the intramuscular fat infiltration. We further measured the fat mass and lean body mass by EchoMRI in 6–7 month-old B10, mdx and mdx:lipin1^Tg/0^ mice (Fig. 6C–G). Mdx mice had substantially reduced body fat and increased lean body mass relative to B10 mice. Increasing lipin1 expression levels in mdx: lipin1^Tg/0^ mice did not affect fat, lean mass, body fat to body weight ratios, and lean body weight to body weight ratios (Fig. 6C–G), suggesting lipin1 restoration will not lead to the risk for the development of obesity.

The overexpression of lipin1 has also been identified in situ in prostate cancer and triple negative breast cancer [46, 47]. However, we did not detect any tumor formation in mdx:lipin1^Tg/0^ mice or in the muscle-specific lipin1 transgenic mice.

### Increasing lipin1 levels via intramuscular (IM) injection of AAV1-lipin1 significantly reduced centrally nucleated muscle fibers in mdx mice

To further verify the effects of lipin1 restoration on muscle phenotype, we used a gene delivery approach. To rule out the potential effects of AAV1 vector alone on the pathology of mdx muscles, we injected AAV1-GFP (5 × 10^10^ viral genome particles (vg)/mouse) or saline into gastrocnemius of mdx mice. One-month post-injection, the effect of AAV1 vector on muscle pathology was evaluated by H&E staining, or immunostaining with antibodies against GFP (Supplemental Fig. 1A). Consistent with previous study [48], we found that AAV1 vector administration did not affect muscle fiber central nucleation (Supplemental Fig. 1B). We also evaluated the consequence of AAV vector or saline injection on muscle damage in mdx mice via EBD staining, and we did not observe any difference between AAV vector or saline injection (Supplemental Fig. 1C). Moreover, protein expression levels of necroptotic markers including RIPK1, RIPK3, and MLKL were assessed by Western blotting analysis in gastrocnemius muscles of B10 and mdx mice treated with either GFP (Supplemental Fig. 1D), or saline (Supplemental Fig. 1E). AAV vector and saline treatment exhibited similar levels of elevation in dystrophic muscle compared to B10 WT control groups. Since we need to perform immunohistochemistry with both green and red fluorescent detection and AAV expressing GFP could interfere with the staining, therefore, we used saline as a control group for AAV1-lipin1 injection in the following studies.

**Fig. 7:**
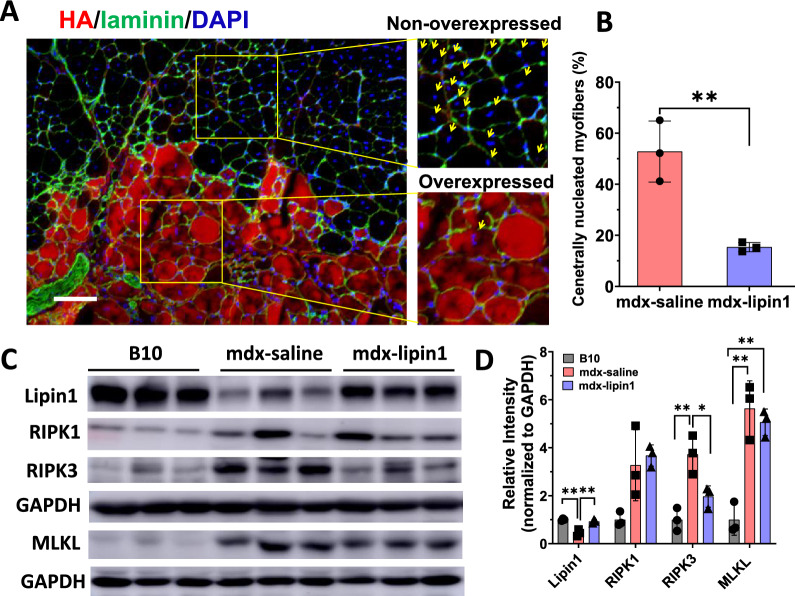
The effects of lipin1 overexpression on dystrophic muscle histology through IM injection. **A** Representative immunofluorescence staining of gastrocnemius muscle from mdx mice 1-month intramuscular post-injection of AAV1-lipin1. Lipin1 was detected by anti-HA antibody (red) and laminin by anti-laminin antibody (green). DAPI was used to stain nucleus (blue). Arrows in the right panels indicate centrally nucleated myofibers, which are characteristic of muscle regeneration (scale bar = 100 μm). **B** The percentage of centrally nucleated myofibers in the lipin1 overexpressed regions compared with the non-overexpressed regions (n = 3 mice/group, **p < 0.01). **C** Western blot and **D** quantification analysis of necroptotic markers in mdx mice treated with either saline or AAV-lipin1 (n = 3 mice/group, *p < 0.05; **p < 0.01)

We injected HA-tagged AAV1-lipin1 (5 × 10^10^ vg/mouse) into the gastrocnemius of 2-month-old male mdx mice. One-month post-injection, the gastrocnemius muscles were harvested for immunofluorescence. Mdx muscle fibers without lipin1 restoration exhibited central nuclei in 55.7% of myofibers (arrows), indicative of muscle degeneration-regeneration (Fig. 7A, B). In lipin1-restored regions, the percentage of centrally nucleated fibers was markedly reduced to 13%. Myofiber sizes were noticeably larger when compared with those in non-restored regions and the mean myofiber cross-sectional area was increased from 1095 μm^2^ in non-restored regions to 1915 μm^2^ in restored regions.

To verify the prevention effects of lipin1 restoration on muscle degeneration, we assessed these necroptosis markers in gastrocnemius of 3-month-old mdx male mice treated with either AAV1-lipin1 or saline via intramuscular injection 1-month post-injection. Gastrocnemius from B10 mice were used as controls. Consistent with what we observed in mdx:lipin1^Tg/0^ mice, AAV1-lipin1 administration through intramuscular injection inhibited the protein expression levels of RIPK1, RIPK3, and MLKL by 50%, 35%, and 35%, respectively (Fig. 7C, D).

### Restoration of lipin1 suppressed necroptosis in cell culture system

**Fig. 8:**
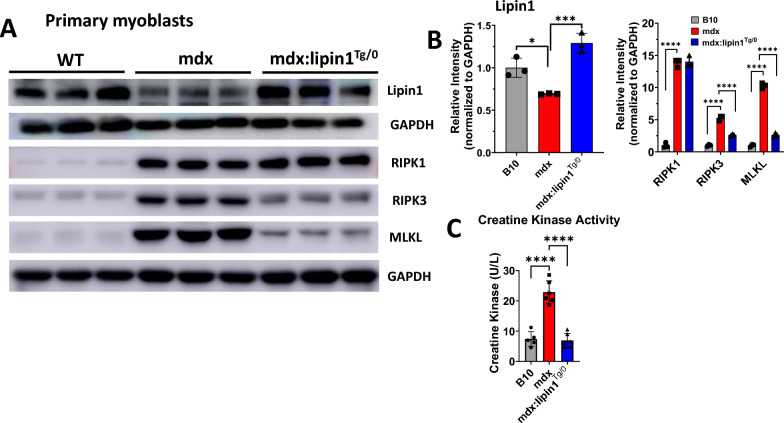
Overexpression of lipin1 in differentiated primary myoblasts isolated from mdx:lipin1^Tg/0^ mice suppressed necrotic cell death compared to differentiated mdx myoblasts. **A** Protein expression levels and **B** quantification analysis of necroptotic makers measured by Western blotting after primary myoblasts isolated from B10, mdx, and mdx:lipin1^Tg/0^ mice followed by differentiation for 6 days. **C** CK levels in the cell culture medium of differentiated B10, mdx, and mdx:lipin1^Tg/0^ primary myoblasts collected at day 4 post-differentiation treatment. *p < 0.05, **p < 0.01; ****p < 0.0001

To elucidate the role of lipin1 in mechanisms underlying the inhibition of necroptosis in dystrophic muscles, we also isolated primary myoblasts from B10 WT, mdx, and mdx:lipin1^Tg/0^ mice. Primary myoblasts isolated from these mice were subjected to myoblast differentiation for 6 days, and protein expression levels of necroptotic markers were measured. We found that primary myoblasts isolated from mdx mice exhibited increased RIPK3 and MLKL by 5.27-fold (*p* < *0.0001*) and 10.3-fold (*p* < *0.0001*), respectively, compared to differentiated WT myoblast controls (Fig. 8A, B). In contrast, differentiated primary myoblasts isolated from mdx:lipin1^Tg/0^ suppressed elevated RIPK3 and MLKL by 50% (*p* < *0.0001*) and 74% (*p* < *0.0001*), respectively, suggesting that lipin1 deficiency led to upregulation of necroptosis, whereas restoring lipin1 expression ameliorated muscular degenerative phenotype by suppressing necroptosis.

To further identify whether lipin1 inhibits necroptosis, we measured CK levels in the cell culture medium (day 4) of differentiated primary myoblasts isolated from mdx, mdx:lipin1^Tg/0^ mice, and B10 WT controls (Fig. 8C). We found that CK levels were increased by 4.3-fold in mdx myotubes, but were downregulated by 77% in mdx:lipin1^Tg/0^ myotubes in the medium, suggesting that restoration of lipin1 improved plasma membrane integrity and inhibited necroptosis in muscle cells.

## Discussion

In this study, we aimed to examine the effects of lipin1 restoration on the improvement of dystrophic phenotypes. We restored lipin1 expression in dystrophic muscles of mdx mice using two different approaches including muscle-specific mdx:lipin1 transgenic mice and AAV gene delivery. Our data clearly showed that lipin1 restoration substantially improved membrane integrity indicated by reduced EBD staining, inhibited myofiber death, inflammation, and fibrosis, and improved in situ isometric and eccentric muscle force generation, and whole animal motor function.

A major problem that leads to DMD symptoms is compromised cellular membranes caused by dystrophin deficiency. Loss of membrane integrity is associated with increased calcium influx, muscle degeneration, and loss of myofibers, which is mainly mediated by a necrotic cell death process. When the disease progresses, a replacement of the myofibers by fibrosis is associated with muscle wasting and weakness. Our results suggest that lipin1 restoration leads to improved membrane integrity, reduced muscle damage, suppressed inflammation, prevented muscle degeneration, attenuated deleterious fibrosis, and reduced serum CK levels. This is consistent with our previous studies that knockout of lipin1 in skeletal muscle leads to impaired membrane integrity detected by elevated EBD uptake and increased CK levels [15]. Further knockout of lipin1 in dystrophic muscle results in more severe muscle damage. In addition, using primary myocytes in a cell culture system, we also found that lipin1 deficiency causes sarcolemma leakage represented by elevated signal of calcium impermeable dye (Fluo-4) in lipin1 deficient myotubes, and increased CK level in cell culture media, and elevated necroptotic markers [16]. In contrast, increasing lipin1 expression levels by AAV1-lipin1 treatment in differentiated lipin1-deficient primary myoblasts inhibits protein expression levels of necroptotic markers. Dystrophin deficiency has been shown previously to lead to a concomitant loss of protein expression of DAPC proteins. Here, we demonstrated that increasing lipin1 expression levels in dystrophic muscle prevented muscle damage, rescued the protein expression levels of α-sarcoglycan, dystroglycan, and nNOS, and promoted sarcolemmal stability.

To determine whether lipin1 restoration in dystrophic muscle could affect muscle regeneration, we measured the protein expression levels of pax7 and myoD, and found that pax7 and myoD were highly elevated in dystrophic muscle compared to WT controls due to continuous muscle degeneration. Consistently, ERK was activated in dystrophic muscle to promote cell proliferation and muscle regeneration. In contrast, mdx:lipin1^Tg/0^ muscle had reduced muscle regeneration due to much less muscle damage, and therefore manifested lower regeneration compared to mdx mice. In our previous studies, we found that lipin1 deficiency leads to prolonged regeneration [16, 25]. Therefore, we expect that restoration of lipin1 could be beneficial for muscle regeneration. Based on the current staining and Western blotting data, it would be difficult to define the role of lipin1 in muscle regeneration. Further studies will be needed to define the role of lipin1 in muscle regeneration by cardiotoxin or BaCl_2_-induced muscle injury, and using satellite cell-specific lipin1 knockout mice.

Lipin1 has dual functions acting as a PAP enzyme in the triglyceride synthesis pathway and as a transcriptional co-regulatory activity [49]. Studies demonstrate that the skeletal muscle of mdx mice and of patients with DMD have altered phospholipid profiles, specifically showing reduced PC levels [50, 51], and decreased PC levels are suggested to be an early event in the muscle degeneration-regeneration process [50–52]. It is likely that increasing lipin1 levels corrected the decreased DAG and PC levels in the mdx muscle. In addition to its role in phospholipid biosynthesis, DAG is also a signaling molecule. Our recent publication identified a previously unknown role of lipin1 in promoting myocyte enhancer factor 2c (MEF2c) transcriptional activity through DAG signaling [24]. MEF2c regulates skeletal muscle cell integrity by controlling the expression of a number of sarcolemma genes [53]. In the liver, lipin1 has been shown to interact with PPARγ coactivator-1α (PGC-1α) to regulate the expression of genes involved in fatty acid oxidation [49]. PGC-1α overexpression is known to protect against dystrophy in mdx mice [54, 55]. However, our prior research has suggested that mice expressing a truncated allele of lipin1 that lacked PAP activity, but with preserved transcriptional coregulatory function, exhibit skeletal myopathy [20]. Furthermore, at least one pathogenic human LPIN1 mutation (p.Arg725His) results in lipin1 protein that is well expressed and retains its transcriptional regulatory function, but is deficient in PAP activity [56]. This suggests that lipin1 PAP activity is critical for its effects on myocyte injury, but further study is needed to elucidate the underlying mechanism of lipin1 in maintaining membrane integrity.

Patients with DMD usually lose the capability to walk as early as 7 years of age and will have to rely on wheelchairs by the age of 10–12 years [57]. It has been shown that reduced force production in DMD might result from decreased muscle fiber mass/number due to myofiber death, and concomitant to the increased tissue stiffness due to fibrosis and fat infiltration [58]. Dystrophic muscles have been shown to have increased deposition of collagen which leads to increased muscle size [59, 60]. This increase in size is called pseudo-hypertrophy which, functionally, does not generate more force [61–64]. Moreover, the increased non-muscle material may hinder muscle function. In addition, other factors may contribute to reduced force generation in dystrophic muscles such as increased percentage of regenerated fibers, disrupted dystrophin-associated protein complex, disconnection between force transducing molecules, and sarcolemma [57, 65–67]. The reduced force production contributes to muscle weakness and compromises the patient’s quality of life. In contrast, we found that restoration of lipin1 in mdx mice led to improved muscle force production including contractile force and eccentric force. Moreover, increasing lipin1 expression levels improved muscle strength measured by the grip strength test and force production measurement, and increased endurance measured by the treadmill exhaustion test. These muscle function improvements could be due to attenuated histopathology by lipin1 restoration including strengthened sarcolemma integrity, inhibited dystrophic necroptosis, suppressed inflammation, and prevented myofiber fibrosis. Nevertheless, improving muscle force and performance would be expected to greatly improve patients’ quality of life.

We also explored the potential adverse effects of lipin1 restoration in dystrophic muscle. Lipin1 catalyzes the conversion of phosphatidic acid to diacylglycerol which is the penultimate step of triglyceride synthesis [68]. Using Oil Red O and BODIPY staining, we did not observe lipid droplets formation in gastrocnemius muscle of mdx:lipin1^Tg/0^ mice suggesting that increasing lipin1 expression levels in dystrophic muscle did not lead to excessive intramyocellular lipid deposition. Consistently, we did not detect alteration in protein expression levels of perilipin 2 in mdx:lipin1^Tg/0^ compared to mdx mice. We also compared fat mass distribution between mdx and mdx:lipin1^Tg/0^ mice, and found that restoration of lipin1 in dystrophic muscle did not affect the fat and lean body mass suggesting that restoring the physiological level of the lipin1 will not lead to the risk for the development of obesity. Moreover, previous studies also showed that overexpression of lipin1 was detected in situ in prostate cancer and in triple negative breast cancer cells [46, 47, 69], whereas lipin1 knockdown reduces the growth of tumor xenograft [47], while barely affecting normal control cells. We did not detect any tumor development in our mdx:lipin1^Tg/0^ mice, even in muscle-specific lipin1 transgenic mice. These data suggest that restoring the physiological level of the lipin1 in mdx:lipin1^Tg/0^ mice did not show any obvious adverse effects, which demonstrates the safety of lipin1 restoration.

Overall, our results suggest that restoration of lipin1 expression or activity could be a potential therapeutic target that may help stabilize the sarcolemma and counter muscle degeneration and necrosis. The lipin1-mediated sarcolemma stability represents a novel pathway for DMD treatment. We anticipate that lipin1 gene delivery will have broad applicability for DMD, irrespective of the dystrophin mutations. In addition to having therapeutic efficacy on its own, the unique action of lipin1 could synergize with these other gene delivery strategies, in combination with therapies aimed at restoring dystrophin by mini-dystrophin, micro-dystrophin, and antisense oligonucleotide-mediated exon skipping. However, the exploratory results reported herein should be considered in the light of some limitations. Firstly, An AAV-based systemic delivery method will be needed to restore lipin1 as a therapeutic tool. We will evaluate the efficiency of systemic intravenous administration of AAV-lipin1 to mdx mice. Secondly, we will investigate in deep the mechanisms by which lipin1 restoration ameliorates the dystrophic phenotype of mdx mice. In the future study, we should investigate effectiveness, possible adverse effect and long-term safety of different expression levels of lipin1 as a potential therapy for DMD.

### Supplementary Information

Supplementary Material 1. Supplemental Fig. 1 AAV1-GFP treatment did not affect dystrophic phenotypes in gastrocnemius of mdx mice.

## Data Availability

The datasets used and/or analyzed during the current study are available from the corresponding author on reasonable request.

## Abbreviations

EBD: Evans blue dye
PA: Phosphatidic acid
PAP: Phosphatidic acid phosphatase
DAG: Diacylglycerol
WT: Wild-type
eMyHC: Embryonic myosin heavy chain
RIPK: Receptor-interacting serine/threonine-protein kinase
MLKL: Mixed-lineage kinase domain-like protein
OCT: Optimal cutting temperature
i.p.: Intraperitoneal
IM: Intramuscular
C.Cas 3: Cleaved caspase 3
