# Supplementary material for: Exploring lipin1 as a promising therapeutic target for the treatment of Duchenne muscular dystrophy
Source: J Transl Med. 2024 Jul 16;22:664. doi: 10.1186/s12967-024-05494-z (PMC11253568; doi:10.1186/s12967-024-05494-z)
Supplement: Supplementary file 1 — Supplementary Material 1. Supplemental Fig. 1 AAV1-GFP treatment did not affect dystrophic phenotypes in gastrocnemius of mdx mice. [file 12967_2024_5494_MOESM1_ESM.pdf]

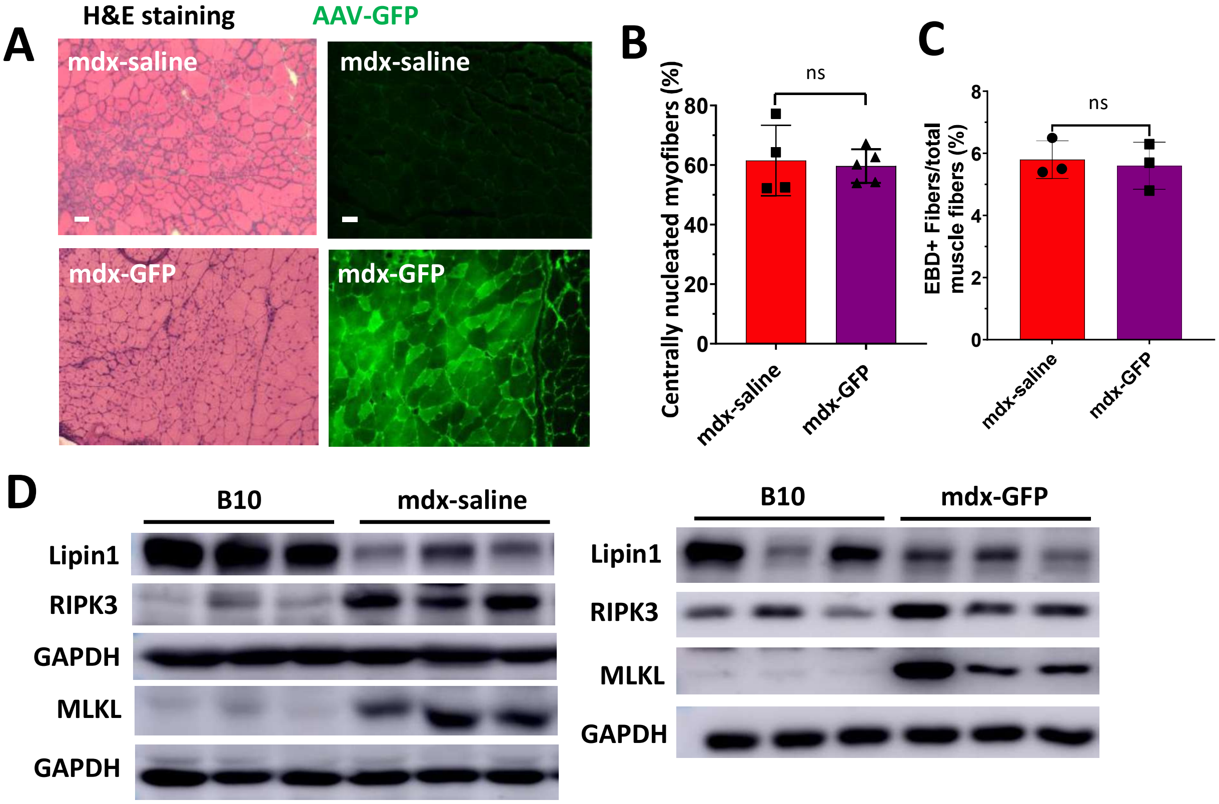

Supplemental Fig. 1 AAV1-GFP treatment did not affect dystrophic phenotypes in gastrocnemius of mdx mice. ***(A)*** Representative H&E staining and GFP expression of gastrocnemius muscle sections of mdx mice treated with either AAV1-GFP or saline. ***(B)*** Quantification analysis of centrally nucleated fibers in mdx mice treated with either AAV1-GFP or saline. ***(C)*** Muscle damage was assessed by EBD staining in mdx mice treated with either AAV1-GFP or saline. Western blotting analysis of necroptotic markers in mdx mice treated with either ***(D)*** AAV1-GFP or ***(E)*** saline compared to B10 WT mice. Scale bar = 50 um.
